# Effect of lifestyle intervention on the reproductive endocrine profile in women with polycystic ovarian syndrome: a systematic review and meta-analysis

**DOI:** 10.1530/EC-14-0010

**Published:** 2014-02-28

**Authors:** Liza Haqq, James McFarlane, Gudrun Dieberg, Neil Smart

**Affiliations:** 1 School of Science and Technology, University of New EnglandArmidale, New South Wales, 2351Australia

**Keywords:** exercise, follicle-stimulating hormone, luteinizing hormone, insulin resistance, female reproduction, polycystic ovarian syndrome

## Abstract

Polycystic ovarian syndrome (PCOS) affects 18–22% of women at reproductive age. We conducted a systematic review and meta-analysis evaluating the expected benefits of lifestyle (exercise plus diet) interventions on the reproductive endocrine profile in women with PCOS. Potential studies were identified by systematically searching PubMed, CINAHL and the Cochrane Controlled Trials Registry (1966–April 30, 2013) systematically using key concepts of PCOS. Significant improvements were seen in women receiving lifestyle intervention vs usual care in follicle-stimulating hormone (FSH) levels, mean difference (MD) 0.39 IU/l (95% CI 0.09 to 0.70, *P*=0.01), sex hormone-binding globulin (SHBG) levels, MD 2.37 nmol/l (95% CI 1.27 to 3.47, *P*<0.0001), total testosterone levels, MD −0.13 nmol/l (95% CI −0.22 to −0.03, *P*=0.008), androstenedione levels, MD −0.09 ng/dl (95% CI −0.15 to −0.03, *P*=0.005), free androgen index (FAI) levels, MD −1.64 (95% CI −2.94 to −0.35, *P*=0.01) and Ferriman–Gallwey (FG) score, MD −1.01 (95% CI −1.54 to −0.48, *P*=0.0002). Significant improvements were also observed in women who received exercise-alone intervention vs usual care in FSH levels, MD 0.42 IU/l (95% CI 0.11 to 0.73, *P*=0.009), SHBG levels, MD 3.42 nmol/l (95% CI 0.11 to 6.73, *P*=0.04), total testosterone levels, MD −0.16 nmol/l (95% CI −0.29 to −0.04, *P*=0.01), androstenedione levels, MD −0.09 ng/dl (95% CI −0.16 to −0.03, *P*=0.004) and FG score, MD −1.13 (95% CI −1.88 to −0.38, *P*=0.003). Our analyses suggest that lifestyle (diet and exercise) intervention improves levels of FSH, SHBG, total testosterone, androstenedione and FAI, and FG score in women with PCOS.

## Introduction

Polycystic ovarian syndrome (PCOS) is a heterogeneous endocrine disorder, affecting 18–22% of reproductive-age women (1). PCOS was first reported in 1935 by Stein & Leventhal (2) and is characterised by clinical or biochemical hyperandrogenism (clinical manifestations are hirsutism, android alopecia and acne), oligo/amenorrhoea (infrequent or no menstruation), polycystic ovaries and infertility or reduced fertility (3, 4). Often women with PCOS are obese, which contributes to insulin resistance and hyperinsulinaemia, but these two features are also present in lean women with PCOS (5, 6). Hormonal manifestations include elevated levels of androgens (testosterone, DHEA and androstenedione), oestrogens and prolactin. Occasionally, thyroid-stimulating hormone levels are also lower leading to hypothyroidism (7). Most women with PCOS have elevated luteinising hormone (LH) levels and reduced follicle-stimulating hormone (FSH) levels particularly during the follicular phase of the menstrual cycle (8). The elevated LH level probably increases the follicular androgen concentrations leading to follicular arrest and the reduced FSH concentrations lead to an accumulation of small follicles (9). The resultant oestrogen environment alters the hypothalamic release of gonadotrophin-releasing hormone and leads to an increase in LH secretion and suppression of FSH secretion by the pituitary (8, 10). This altered LH:FSH ratio is used as a diagnostic criterion for this condition, but it is not universally present (11).

The levels of sex hormone-binding globulin (SHBG), the primary plasma transport system which controls the availability of androgens, are reduced in women with PCOS leading to an increase in free testosterone levels contributing to the free androgen index (FAI) (12). Owing to the effects of insulin on hepatic SHBG production, insulin insensitivity may affect ovulation and fertility. Dyslipidaemia, increased insulin levels, obesity, hypertension, impaired glucose tolerance and insulin-induced metabolic syndrome are also the risk factors that can predispose women with PCOS to cardiovascular disease and type 2 diabetes mellitus (6).

A systematic review was completed in 2011 by Harrison *et al*. (13), but presumably as insufficient data were available at that time, these authors did not conduct data pooled analyses. A systematic review and subsequent meta-analyses were conducted by Moran *et al*. (14), but these analyses included only six published studies with slightly different inclusion/exclusion criteria. However, our work provides a greater number of hormonal analyses when compared with Moran's work (14). We therefore conducted a systematic review and meta-analysis, and the primary aim was to evaluate the expected benefits of exercise training and dietary interventions on a range of endocrinal outcomes in women with PCOS.

## Subjects and methods

### Search strategy

Potential studies were identified by conducting a systematic search using PubMed (www.ncbi.nlm.nih.gov/pubmed; 1966–April 30, 2013). CINAHL and the Cochrane Controlled Trials Registry were also used for the search (1966–April 30, 2013). The search strategy included the key concepts of PCOS, dietary therapy, lifestyle therapy and exercise training. These were combined with a sensitive search strategy to identify randomised controlled trials. Reference lists of papers found were scrutinised for new references. All identified papers were assessed independently by two reviewers (N Smart and L Haqq). The search for published papers continued up until April 30, 2013.

### Inclusions

Randomised, controlled trials of exercise-alone or lifestyle (exercise plus diet) intervention in people with PCOS were included. There were no language restrictions.

### Exclusions

Animal studies, review papers and non-randomised controlled trials were excluded. Studies that did not have desired outcome measures or used non-PCOS participants in exercise or lifestyle (exercise plus diet) interventions or usual care groups were excluded. Several authors were contacted and they provided missing data, these data were used in the analyses. Incomplete data or data from an already included study were excluded. Studies using interventions other than lifestyle (e.g. electro-acupuncture and ultrasound) were excluded.

### Studies included in the review

**Figure 1:**
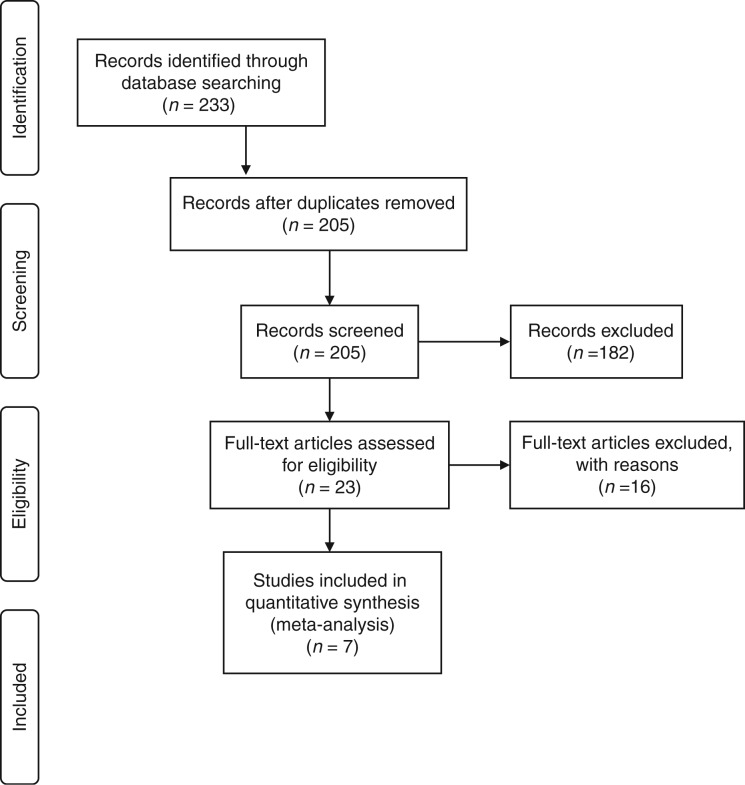
Consort statement.

Our initial search identified 201 manuscripts, and looking into the latest editions of relevant journals, a further 32 manuscripts were identified. Out of 233 studies, 28 were excluded at first inspection as duplicates, and 182 were removed after reading titles or abstracts, leaving 23 studies of which 16 studies were excluded for various reasons (including five studies which did not provide data that could be included in our analysis), leaving seven studies for final analysis (see consort statement, Fig. 1).

### Data synthesis and outcome measures

Our lifestyle intervention groups were defined as exercise-alone or exercise-plus-diet groups. Our definition of usual care (comparator) groups could include sedentary control, placebo, diet only or medication. Analyses were conducted only on intervention vs comparator 1 (see Table 1). Information regarding all outcome measures was archived in a database. The outcome measures comprised hormones and hormone ratios, including LH, FSH, SHBG, total testosterone, measured free testosterone, androstenedione, FAI, LH:FSH ratio, oestradiol (E_2_) and Ferriman–Gallwey (FG) score.

### Statistical analysis

Meta-analyses were completed for continuous data using the change in the mean and s.d. of outcome measures, as we did not wish to assume that randomisation would adjust for baseline imbalance. Change in post-intervention mean was calculated by subtracting baseline from post-intervention values. Change in the s.d. of post-intervention outcomes was calculated using RevMan 5.0 (Nordic Cochrane Centre, Copenhagen, Denmark). Data required were either i) 95% CI data for pre–post intervention change for each group or, when this was unavailable, ii) actual *P* values for pre–post intervention change for each group or, if only the level of statistical significance was available and iii) we used default *P* values (e.g. *P*<0.05 becomes *P*=0.049, *P*<0.01 becomes *P*=0.0099 and *P*=not significant becomes *P*=0.05). A random-effects inverse variance was used with the effects measure of mean difference (MD). Heterogeneity was quantified using Cochran's *Q* test (15). Sensitivity analyses were conducted by removing studies of lifestyle intervention, leaving studies of exercise only, for the outcomes, such as FSH, SHBG, total testosterone, androstenedione, FAI, E_2_, LH:FSH ratio and FG score. The purpose of sensitivity analyses was to compare the effect sizes of exercise alone with exercise plus diet. Egger plots (16) were produced to assess the risk of publication bias.

Study quality was assessed using a modified PEDro (17) score (out of 9 maximum scores) as blinding participants is difficult in lifestyle studies. We used a 5% level of significance and 95% CIs; figures were produced using RevMan 5.0.

## Results

Our analyses included data from seven studies (3, 18, 19, 20, 21, 22, 23), which yielded data on 206 women with PCOS. In three studies, the mean BMI indicated that the participants were obese, three studies indicated that women were overweight and in one study this was unclear. The mean age of participants in all but one study was 21–32 years of age. Details about the number of participants, duration of studies and withdrawals for included studies can be seen in Table 1. Table 2 contains detailed descriptions of all interventions and comparator groups. Details about baseline characteristics of participants in the included studies can be seen in Table 3. Details about the excluded randomised, controlled, trials (4, 11, 24, 25, 26, 27, 28, 29, 30, 31, 32, 33, 34, 35, 36, 37) can be seen in Table 4.

### Hormonal parameters

LH levels were not significantly different for women in lifestyle intervention vs usual care groups, with a MD of 0.99 IU/l (95% CI −0.11 to 2.09, *P*=0.08). Moreover, LH levels were also not significantly different for women in exercise vs usual care groups, with an MD of 0.51 IU/l (95% CI −1.11 to 2.13, *P*=0.54).

**Figure 2:**
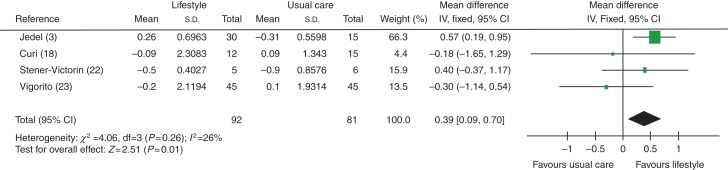
Change in FSH levels in lifestyle intervention vs usual care groups.

FSH levels were found to be improved (higher) in women in lifestyle intervention vs usual care groups, with an MD of 0.39 IU/l (95% CI 0.09 to 0.70, *P*=0.01), see Fig. 2.

**Figure 3:**
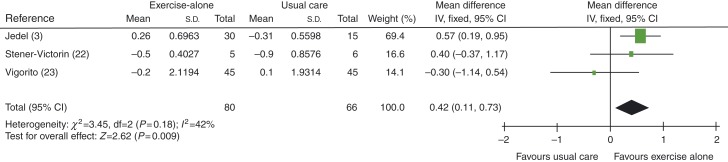
Change in FSH levels in exercise-alone vs usual care groups.

When studies using exercise plus diet were removed to distinguish between exercise-alone and exercise-plus-diet groups, FSH levels were found to be improved in exercise-alone group, with an MD of 0.42 IU/l (95% CI 0.11 to 0.73, *P*=0.009), see Fig. 3.

**Figure 4:**
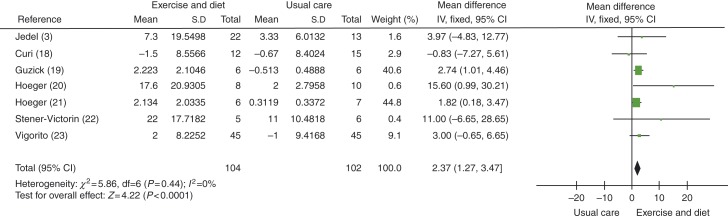
Change in SHBG levels in lifestyle intervention vs usual care groups.

SHBG levels were found to be improved in women in lifestyle intervention vs usual care groups, with an MD of 2.37 nmol/l (95% CI 1.27 to 3.47, *P*<0.0001), see Fig. 4.

**Figure 5:**
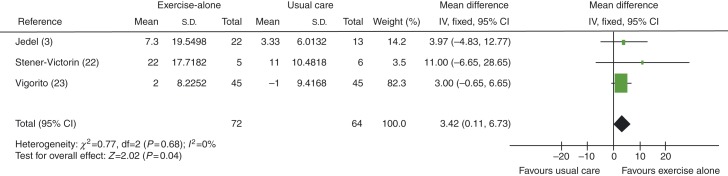
Change in SHBG levels in exercise-alone vs usual care groups.

When studies using exercise plus diet were removed to distinguish between exercise-alone and exercise-plus-diet groups, SHBG levels showed greater improvement in exercise-alone groups, with an MD of 3.42 nmol/l (95% CI 0.11 to 6.73, *P*=0.04), see Fig. 5.

**Figure 6:**
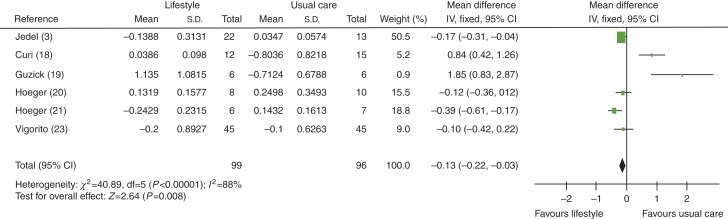
Change in total testosterone levels in lifestyle intervention vs usual care groups.

Measures of total testosterone levels were found to be improved significantly (lowered) in lifestyle intervention vs usual care groups, with an MD of −0.13 nmol/l (95% CI −0.22 to −0.03, *P*=0.008), see Fig. 6.

**Figure 7:**
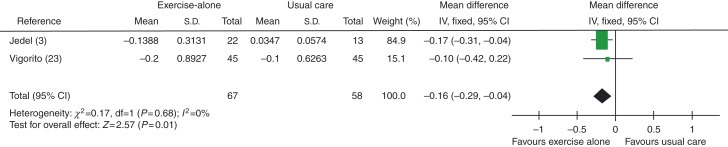
Change in total testosterone levels in exercise-alone vs usual care groups.

When studies using exercise plus diet were removed to distinguish between exercise-alone and exercise-plus-diet groups, total testosterone levels were found to be improved significantly (lowered) in exercise-alone group, with an MD of −0.16 nmol/l (95% CI −0.29 to −0.04, *P*=0.01), see Fig. 7.

Measured free testosterone levels were found to be slightly higher in women in exercise-alone vs usual care groups, with an MD of 0.66 nmol/l (95% CI 0.13 to 1.19, *P*=0.01).

**Figure 8:**
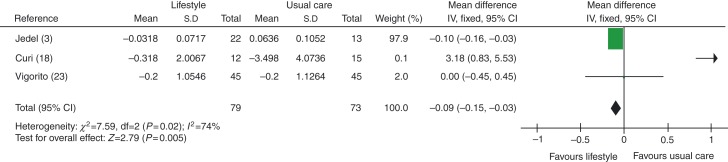
Change in androstenedione levels in lifestyle intervention vs usual care groups.

Androstenedione/4-DION levels were found to be improved (lower) in women in lifestyle intervention vs usual care groups, MD −0.09 ng/l (95% CI −0.15 to −0.03, *P*=0.005), see Fig. 8.

**Figure 9:**
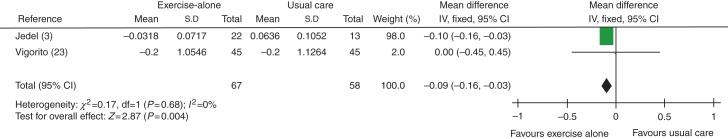
Change in androstenedione levels in exercise-alone vs usual care groups.

When studies using exercise plus diet were removed to distinguish between exercise-alone and exercise-plus-diet groups, androstenedione levels were found to be similar in exercise-alone group and the lifestyle intervention group, with an MD of −0.09 ng/dl (95% CI −0.16 to −0.03, *P*=0.004), see Fig. 9.

**Figure 10:**
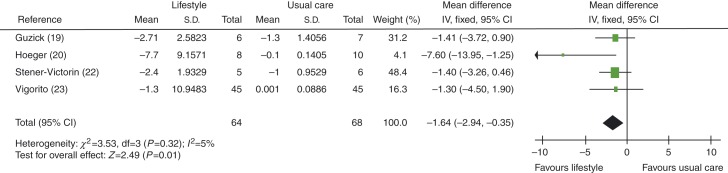
Change in FAI levels in lifestyle intervention vs usual care groups.

FAI levels were found to be improved significantly (lower) in women in the lifestyle intervention vs usual care groups, with an MD of −1.64 (95% CI −2.94 to −0.35, *P*=0.01), see Fig. 10. When studies using exercise plus diet were removed to distinguish between exercise-alone and exercise-plus-diet groups, FAI levels were not significantly decreased in exercise-alone group, with an MD of −1.38 (95% CI −2.98 to 0.23, *P*=0.09).

**Figure 11:**
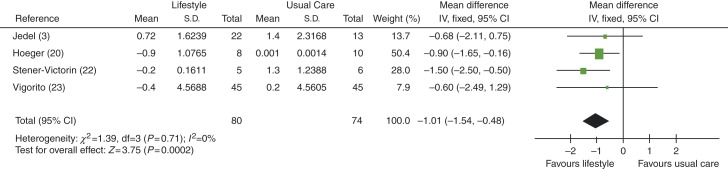
Change in FG score in lifestyle intervention vs usual care groups.

FG score was found to be improved (lower) in women in lifestyle intervention vs usual care groups, with an MD of −1.01 (95% CI −1.54 to −0.48, *P*=0.0002), see Fig. 11.

**Figure 12:**
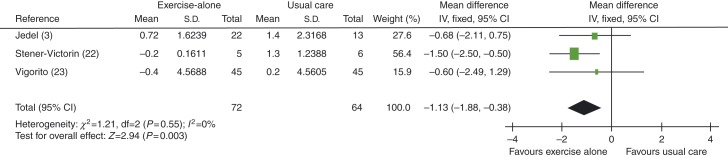
Change in FG score in exercise-alone vs usual care groups.

When studies using exercise plus diet were removed to distinguish between exercise-alone and exercise-plus-diet groups, FG score was found to be improved in women in exercise-alone vs usual care groups, with an MD of −1.13 (95% CI −1.88 to −0.38, *P*=0.003), see Fig. 12.

The change in E_2_ levels with an MD of −8.51 pmol/l (95% CI −25.2 to 8.15, *P*=0.32) and LH:FSH ratio with an MD of 0.01 (95% CI −0.20 to 0.22, *P*=0.94) were not significant in women in exercise-alone vs usual care groups.

## Study quality

In terms of study quality, the median score was 7, with one study scoring 6, three studies scoring 7, two studies scoring 8 and one study scoring 9, using a modified PEDro scale (out of 9). Details about the scores and PEDro scale are given in Table 5. Egger plots showed little or no evidence of publication bias.

## Discussion

This study is an update from a 2011 meta-analysis examining the effectiveness of lifestyle intervention on PCOS (14). We examined the effects of lifestyle intervention on endocrinal parameters and the FG score. Unlike the previous meta-analysis (14), we took the approach of conducting analyses on combined exercise and dietary (lifestyle) interventions and also sensitivity analyses consisting of exercise-alone intervention groups; the comparator for both main and sensitivity analyses were usual care groups. Our findings suggest that levels of FSH, SHBG, total testosterone, androstenedione and FAI, and FG score were found to be improved in response to lifestyle intervention, while levels of FSH, SHBG, total testosterone and androstenedione, and FG score were found to be improved with exercise alone.

Our findings suggest that lifestyle intervention is beneficial for improving FSH profile. Previous work has not clearly defined the effects of exercise or diet on FSH levels in women with PCOS. The most significant improvement (increase) in SHBG levels (narrowest CI) was observed in the combined exercise and dietary intervention vs usual care. Although the effect size of improvement in SHBG was larger for exercise-alone group compared with usual care groups, the wider CI limits the inferences that can be drawn from this finding. This leads us to conclude that exercise, either in combination with dietary treatment or in isolation, improves the SHBG profile. The hypothesis that, in combination with appropriate dietary intervention, there is an improvement in SHBG levels has been suggested previously (11).

Total testosterone levels were reduced in both the exercise-alone and lifestyle intervention vs usual care groups in our analyses. Our findings reassert the results of previous work which suggest that reductions in total testosterone levels are achieved by lifestyle intervention (3, 23).

Our analyses demonstrate that lifestyle intervention vs usual care groups showed improvement (reduced) in FAI, while there was no change in the exercise-alone vs usual care groups. Our findings therefore suggest that lifestyle intervention is the optimal therapy for eliciting beneficial effects; previous work has also suggested this to be the case (38).

FAI is the total testosterone level divided by the SHBG level. In our analyses, levels of FAI decreased, SHBG increased and total testosterone decreased after exercise-plus-diet intervention. While these effects are consistent with the expectations and are self-explanatory, the timing of sample collection could have moderated the findings due to peaks and troughs in these hormones during the ovulatory cycle.

Lifestyle intervention showed a significantly favourable reduction in androstenedione levels. Our findings showed almost identical reductions in androstenedione levels in lifestyle intervention and exercise-alone groups. These findings support the results of previous work suggesting that exercise reduces androstenedione levels (3), while our analyses suggested limited evidence of additional benefit from diet.

Lifestyle intervention or exercise alone showed a significantly favourable improvement in hirsutism (FG score). These findings support those of previous work suggesting that both lifestyle intervention and exercise improve hirsutism (14).

### Limitations

The sample size of this analysis may be underpowered. Analyses in Figs 6 and 8 exhibit moderate to high evidence of between-study heterogeneity, but the majority of our analyses have little or no heterogeneity. As always with exercise training studies, there are small variations in study duration and exercise modality, although these appear to be minimal in this analysis. While not necessarily a limitation, we chose to modify the PEDro scale to assess the study quality as all studies would have found it impossible to blind participants and investigators to the allocation of exercise training or sedentary control. The Egger plot for testosterone showed minimal evidence of publication bias, while the other Egger plots showed no such evidence of publication bias. It is therefore not probable that unpublished datasets exist for the majority of our outcome measures and the level of significance for testosterone suggests that unpublished data would not change the findings presented here.

### Recommendations for future research

Measures such as testosterone, FSH and LH vary with the stage of the menstrual cycle (stage of follicular development) and additionally are secreted in a pulsatile manner, so caution must be exercised in interpreting these present findings. Not all studies stated whether the timing of blood draws was standardised to each individuals' menstrual and ovulatory cycles; moreover, we believe that multiple samples should be collected to determine true baseline measures of FSH and LH precisely. We suspect that LH has a central role in PCOS management and the failure to detect changes with exercise and dietary interventions is probably a result of poor study design. Future studies should employ more rigorous interpretation of the monthly variations in LH that occur naturally. Other hormones such as growth hormone and insulin-like growth factor 1 together with their binding proteins are influenced by exercise and have an impact on ovarian function and we recommend that these should be included in future studies. Future study should also attempt to compare and evaluate the relative effects of exercise alone vs exercise plus diet.

## Conclusions

Our analyses suggest that lifestyle (diet and exercise) intervention improves the levels of FSH, SHBG, total testosterone, androstenedione and FAI, and FG score in women with PCOS. Exercise alone improved all of these outcomes except FAI and LH; however, given the uncertainty of when the blood samples were taken and the fact that LH levels increase dramatically towards ovulation, further studies are required to elucidate this finding.

## Figures and Tables

**Table 1:** Duration, number of participants, intervention and comparator groups in lifestyle intervention studies.

**Reference**	**Duration of study**	**Total participants** (lifestyle intervention group)	**Withdrawal** (number of people)	**Intervention**	**Comparator 1**	**Comparator 2**	**Comparator 3**
Jedel (3)	16 weeks	84 (22)	25	Lifestyle (exercise only)	Usual care	Low-frequency electro-acupuncture	
Curi (18)	6 months	40 (12)	13	Lifestyle	Metformin		
Guzick (19)	12 weeks	12 (6)	None	Lifestyle	Usual care		
Hoeger (20)	48 weeks	38 (6)	15	Lifestyle and placebo	Placebo	Metformin	Lifestyle and metformin
Hoeger (21)	24 weeks	43 (8)	9	Lifestyle	Placebo	Metformin	Oral contraceptive
Stener-Victorin (22)	16 weeks	20 (5)	None	Lifestyle (exercise only)	Usual care	Low-frequency electro-acupuncture	
Vigorito (23)	3 months	90 (45)	None	Lifestyle (exercise only)	Usual care		

**Table 2:** Diet, exercise training and comparator group characteristics in lifestyle intervention studies.

**Reference, country**	**Diet characteristics**	**Exercise training characteristics**	**Comparator group characteristics**
Jedel (3), Sweden	NA	Brisk walking, cycling or other aerobic exercise at a self-selected pace	No active intervention
	Totally 30 min/session, 3 days/week	
Curi (18), Brazil	To reduce daily intake by 500 kcal Carbohydrate, 50%; fat, 30% and protein, 20%	30 min walk, three self-weight resistance exercises (squats, sit-ups, push-ups and hamstring stretches) for 10 min, warm-up and cool-down with each session	Metformin, 850 mg metformin capsule (Dimefor, Farmoquimica Co., Rio de Janerio, Brazil) orally twice a day for 6 months
	Totally 40–45 min/session, frequency of exercise training unclear
Guzick (19), USA	Low-calorie diet – 400 kcal of lean meat, fish or fowl and a liquid formula (Optifast) for occasional meals. Multivitamin, calcium and potassium supplement	Walking with an energy expenditure of ∼1050 kJ/week initially, progressing to 4200 kJ/week or 10 miles of walking/week	Untreated controls
5 days/week – total duration of exercise session is unclear	
Hoeger (20), USA	To reduce daily intake by 500 kcal	Exercise sessions were not monitored	Placebo capsules twice a day
		Exercise session of 30 min/day with moderate- to high-intensity exercises	
Frequency of exercise training unclear	
Hoeger (21), USA	To reduce daily intake by 500–1000 kcal/day	Individualised exercise programmes with 150 min of exercise/week	Placebo only, orally twice a day
	Carbohydrates, 50%; fat, 25% and protein, 25%	Frequency of exercise training unclear	
Placebo orally twice a day
Stener-Victorin (22), Sweden	NA	Brisk walking, cycling or other aerobic exercise at a pace described as ‘faster than normal walking but at a pace that could be sustained for 30 min’	Untreated controls
Totally 30 min/session, 3 days/week	
Vigorito (23), Italy	NA	Structured exercise training on a bicycle ergometer with a target of 60–70% VO_2_ max. Each session was preceded by 5 min of warm-up and 5 min of cool-down	All the PCOS population were counselled for a healthy diet Carbohydrates, 50%; fat, 25% and protein, 25%
		Total duration of exercise session is unclear, exercise frequency is 3 days/week	

**Table 3:** Inter-group range of baseline values for outcome measures.

**Reference**	**Age**	**BMI**	**WHR**	**HOMA**	**FG score**	**FAI**	**LH**	**FSH**
Jedel (3)	29.7–30.2	26.8–29.1	NA	NA	10.1–12.1	NA	7.2–9.2	4.0–4.3
Curi (18)	24.6–26.3	31.1–31.8	NA	3.4–3.9	13.2–15.27	NA	6.8–13.8	4.6–5.1
Guzick (19)	31.2–32.2	NA	0.92–0.95	NA	NA	NA	10.3–11.5	NA
Hoeger (20)	15.4–16.0	34.3–37.8	NA	NA	7.8–12.5	10.8–21.7	NA	NA
Hoeger (21)	27.1–30.4	37.1–40.4	0.89±0.96	NA	NA	6.8–10.9	NA	NA
Stener-Victorin (22)	NA	26.8–28.0	0.8	1.6–2.0	9.5–16.1	5.8–7.2	6.8–13.8	4.6–5.1
Vigorito (23)	21.7–21.9	29.3–29.4	0.84–0.86	NA	11.9–12.1	8.5–8.6	23.5–24.2	10.1–10.5

BMI, body mass index; WHR, waist-hip ratio; HOMA, homeostatic model assessment; FG score, Ferriman-Gallwey score; FAI, free androgen index; LH, luteinizing hormone; FSH, follicle stimulating hormone.

**Table 4:** Excluded randomised, controlled trials.

**Reference**	**Reason for exclusion**
Thomson (4)	Data reported unsuitable for analysis
Bruner (11)	No data reported were included in analysis
Stener-Victorin (22)	Data reported unsuitable for analysis
Brown (24)	Data reported as median values so unsuitable for analysis
Galletly (25)	Both intervention groups exercised, no control group
Karimzadeh (26)	Data reported unsuitable for analysis
Ladson (27, 28)	Both intervention and comparator groups underwent similar exercise training, no control group
Ladson (27, 28)	Duplicate of previous study
Ma (29)	Both intervention and comparator groups underwent similar exercise training, no control group
Moran (30)	Both intervention and comparator groups underwent similar exercise training, no control group
Orio (31)	No randomised, matched controls
Otta (32)	Both intervention and comparator groups underwent similar exercise training, no control group
Palomba (33)	Study conducted for 6 weeks, but data reported for 2 weeks only
Thomson (34)	Data reported unsuitable for analysis
Thomson (36)	Data reported unsuitable for analysis
Nybacka (37)	No data reported were included in analysis

**Table 5:** Assessment of study quality using the modified PEDro scale (maximum score 9). Median score: 7.

**Reference**	**Eligibility criteria specified**	**Random allocation of participants**	**Allocation concealed**	**Groups similar at baseline**	**Assessors blinded**	**Outcome measures assessed in 85% of participants**	**Intention to treat analysis**	**Reporting of between-group statistical comparison**	**Point measures and measures of variability reported**	**Total score out of 9**
Jedel (3)	Yes	Yes	Yes	Yes	Yes	Yes	Yes	Yes	Yes	9
Curi (18)	Yes	Yes	Yes	No	Unclear	Yes	Yes	Yes	Yes	7
Guzick (19)	Yes	Yes	Unclear	No	Unclear	Yes	Yes	Yes	Yes	6
Hoeger (20)	Yes	Yes	Yes	No	Yes	Yes	Yes	Yes	Yes	8
Hoeger (21)	Yes	Yes	Yes	No	Yes	No	Yes	Yes	Yes	7
Stener-Victorin (22)	Yes	Yes	Yes	No	Yes	No	Yes	Yes	Yes	7
Vigorito (23)	Yes	Yes	Yes	No	Yes	Yes	Yes	Yes	Yes	8

## References

[1] March WA, Moore VM, Willson KJ, Phillips DI, Norman RJ, Davies MJ (2010). The prevalence of polycystic ovary syndrome in a community sample assessed under contrasting diagnostic criteria. Human Reproduction.

[2] Stein I, Leventhal ML (1935). Amenorrhea associated with bilateral polycystic ovaries. American Journal of Obstetrics and Gynecology.

[3] Jedel E, Labrie F, Odén A, Holm G, Nilsson L, Janson PO, Lind AK, Ohlsson C, Stener-Victorin E (2011). Impact of electro-acupuncture and physical exercise on hyperandrogenism and oligo/amenorrhea in women with polycystic ovary syndrome: a randomized controlled trial. American Journal of Physiology. Endocrinology and Metabolism.

[4] Thomson RL, Buckley JD, Noakes M, Clifton PM, Norman RJ, Brinkworth GD (2008). The effect of a hypocaloric diet with and without exercise training on body composition, cardiometabolic risk profile, and reproductive function in overweight and obese women with polycystic ovary syndrome. Journal of Clinical Endocrinology and Metabolism.

[5] Moran LJ, Pasquali R, Teede HJ, Hoeger KM, Norman RJ (2009). Treatment of obesity in polycystic ovary syndrome: a position statement of the Androgen Excess and Polycystic Ovary Syndrome Society. Fertility and Sterility.

[6] Cobin R, Futterweit W, Nestler JI, Reaven GM, Jellinger PS, Handelsman Y, Redmond GP, Thatcher SS (2005). American Association of Clinical Endocrinologists position statement on metabolic and cardiovascular consequences of polycystic ovary syndrome. Endocrine Practice.

[7] Rachon D (2012). Differential diagnosis of hyperandrogenism in women with polycystic ovary syndrome. Experimental and Clinical Endocrinology & Diabetes.

[8] Erickson GF, Danforth DR (1995). Ovarian control of follicle development. American Journal of Obstetrics and Gynecology.

[9] Franks S, Stark J, Hardy K (2008). Follicle dynamics and anovulation in polycystic ovary syndrome. Human Reproduction Update.

[10] Danforth DR (1995). Endocrine and paracrine control of oocyte development. American Journal of Obstetrics and Gynecology.

[11] Bruner B, Chad K, Chizen D (2006). Effects of exercise and nutritional counseling in women with polycystic ovary syndrome. Applied Physiology, Nutrition, and Metabolism.

[12] Martínez-García MÁ, Gambineri A, Alpañés M, Sanchón R, Pasquali R, Escobar-Morreale HF (2012). Common variants in the sex hormone-binding globulin gene (SHBG) and polycystic ovary syndrome (PCOS) in Mediterranean women. Human Reproduction.

[13] Harrison CL, Lombard CB, Moran LJ, Teede HJ (2011). Exercise therapy in polycystic ovary syndrome: a systematic review. Human Reproduction Update.

[14] Moran LJ, Hutchison SK, Norman RJ, Teede HJ (2011). Lifestyle changes in women with polycystic ovary syndrome. Cochrane Database of Systematic Reviews.

[15] Higgins JP, Altman DG, Gotzsche PC, Juni P, Moher D, Oxman AD, Savovic J, Schulz KF, Weeks L, Sterne JA, Cochrane Bias Methods Group, Cochrane Statistical Methods Group. (2011). The Cochrane Collaboration's tool for assessing risk of bias in randomised trials. British Medical Journal.

[16] Egger M, Davey Smith G, Schneider M, Minder C (1997). Bias in meta-analysis detected by a simple, graphical test. BMJ.

[17] Maher CG, Sherrington C, Herbert RD, Moseley AM, Elkins M (2003). Reliability of the PEDro scale for rating quality of randomized controlled trials. Physical Therapy.

[18] Curi DD, Fonseca AM, Marcondes JA, Almeida JA, Bagnoli VR, Soares JM, Baracat EC (2012). Metformin versus lifestyle changes in treating women with polycystic ovary syndrome. Gynecological Endocrinology.

[19] Guzick DS, Wing R, Smith D, Berga SL, Winters SJ (1994). Endocrine consequences of weight loss in obese, hyperandrogenic, anovulatory women. Fertility and Sterility.

[20] Hoeger K, Davidson K, Kochman L, Cherry T, Kopin L, Guzick DS (2008). The impact of metformin, oral contraceptives, and lifestyle modification on polycystic ovary syndrome in obese adolescent women in two randomized, placebo-controlled clinical trials. Journal of Clinical Endocrinology and Metabolism.

[21] Hoeger KM, Kochman L, Wixom N, Craig K, Miller RK, Guzick DS (2004). A randomized, 48-week, placebo-controlled trial of intensive lifestyle modification and/or metformin therapy in overweight women with polycystic ovary syndrome: a pilot study. Fertility and Sterility.

[22] Stener-Victorin E, Jedel E, Janson PO, Sverrisdottir YB (2009). Low-frequency electroacupuncture and physical exercise decrease high muscle sympathetic nerve activity in polycystic ovary syndrome. American Journal of Physiology. Regulatory, Integrative and Comparative Physiology.

[23] Vigorito C, Giallauria F, Palomba S, Cascella T, Manguso F, Lucci R, De Lorenzo A, Tafuri D, Lombardi G, Colao A, Orio F (2007). Beneficial effects of a three-month structured exercise training program on cardiopulmonary functional capacity in young women with polycystic ovary syndrome. Journal of Clinical Endocrinology and Metabolism.

[24] Brown AJ, Setji TL, Sanders LL, Lowry KP, Otvos JD, Kraus WE, Svetkey PL (2009). Effects of exercise on lipoprotein particles in women with polycystic ovary syndrome. Medicine and Science in Sports and Exercise.

[25] Galletly C, Moran L, Noakes M, Clifton P, Tomlinson L, Norman R (2007). Psychological benefits of a high-protein, low-carbohydrate diet in obese women with polycystic ovary syndrome – a pilot study. Appetite.

[26] Karimzadeh MA, Javedani M (2010). An assessment of lifestyle modification versus medical treatment with clomiphene citrate, metformin, and clomiphene citrate–metformin in patients with polycystic ovary syndrome. Fertility and Sterility.

[27] Ladson G, Dodson WC, Sweet SD, Archibong AE, Kunselman AR, Demers LM, Lee PA, Williams NI, Coney P, Legro RS (2011). Effects of metformin in adolescents with polycystic ovary syndrome undertaking lifestyle therapy: a pilot randomized double-blind study. Fertility and Sterility.

[28] Ladson G, Dodson WC, Sweet SD, Archibong AE, Kunselman AR, Demers LM, Williams NI, Coney P, Legro RS (2011). The effects of metformin with lifestyle therapy in polycystic ovary syndrome: a randomized double-blind study. Fertility and Sterility.

[29] Ma LK, Jin LN, Yu Q, Xu L (2007). Effect of lifestyle adjustment, metformin and rosiglitazone in polycystic ovary syndrome. Zhonghua Fu Chan Ke Za Zhi.

[30] Moran LJ, Noakes M, Clifton PM, Tomlinson L, Galletly C, Norman RJ (2003). Dietary composition in restoring reproductive and metabolic physiology in overweight women with polycystic ovary syndrome. Journal of Clinical Endocrinology and Metabolism.

[31] Orio F, Giallauria F, Palomba S, Manguso F, Orio M, Tafuri D, Lombardi G, Carmina E, Colao A, Vigorito C (2008). Metabolic and cardiopulmonary effects of detraining after a structured exercise training programme in young PCOS women. Clinical Endocrinology.

[32] Otta CF, Wior M, Iraci GS, Kaplan R, Torres D, Gaido MI, Wyse EP (2010). Clinical, metabolic, and endocrine parameters in response to metformin and lifestyle intervention in women with polycystic ovary syndrome: a randomized, double-blind, and placebo control trial. Gynecological Endocrinology.

[33] Palomba S, Falbo A, Giallauria F, Russo T, Rocca M, Tolino A, Zullo F, Orio F (2010). Six weeks of structured exercise training and hypocaloric diet increases the probability of ovulation after clomiphene citrate in overweight and obese patients with polycystic ovary syndrome: a randomized controlled trial. Human Reproduction.

[34] Thomson RL, Buckley JD, Lim SS, Noakes M, Clifton PM, Norman RJ, Brinkworth GD (2010). Lifestyle management improves quality of life and depression in overweight and obese women with polycystic ovary syndrome. Fertility and Sterility.

[35] Stener-Victorin E, Baghaei F, Holm G, Janson PO, Olivecrona G, Lönn M, Mannerås-Holm L (2012). Effects of acupuncture and exercise on insulin sensitivity, adipose tissue characteristics, and markers of coagulation and fibrinolysis in women with polycystic ovary syndrome: secondary analyses of a randomized controlled trial. Fertility and Sterility.

[36] Thomson RL, Brinkworth GD, Noakes M, Clifton PM, Norman RJ, Buckley JD (2012). The effect of diet and exercise on markers of endothelial function in overweight and obese women with polycystic ovary syndrome. Human Reproduction.

[37] Nybacka Å, Carlström K, Ståhle A, Nyrén S, Hellström PM, Hirschberg AL (2011). Randomized comparison of the influence of dietary management and/or physical exercise on ovarian function and metabolic parameters in overweight women with polycystic ovary syndrome. Fertility and Sterility.

[38] Hoeger KM (2008). Exercise therapy in polycystic ovary syndrome. Seminars in Reproductive Medicine.

